# The Dilemma of Choosing a Reference Character for Measuring Sexual Size Dimorphism, Sexual Body Component Dimorphism, and Character Scaling: Cryptic Dimorphism and Allometry in the Scorpion *Hadrurus arizonensis*

**DOI:** 10.1371/journal.pone.0120392

**Published:** 2015-03-20

**Authors:** Gerad A. Fox, Allen M. Cooper, William K. Hayes

**Affiliations:** Department of Earth and Biological Sciences, School of Medicine, Loma Linda University, Loma Linda, California, United States of America; National and Kapodistrian University of Athens, Faculty of Biology, GREECE

## Abstract

Sexual differences in morphology, ranging from subtle to extravagant, occur commonly in many animal species. These differences can encompass overall body size (sexual size dimorphism, SSD) or the size and/or shape of specific body parts (sexual body component dimorphism, SBCD). Interacting forces of natural and sexual selection shape much of the expression of dimorphism we see, though non-adaptive processes may be involved. Differential scaling of individual features can result when selection favors either exaggerated (positive allometry) or reduced (negative allometry) size during growth. Studies of sexual dimorphism and character scaling rely on multivariate models that ideally use an unbiased reference character as an overall measure of body size. We explored several candidate reference characters in a cryptically dimorphic taxon, *Hadrurus arizonensis*. In this scorpion, essentially every body component among the 16 we examined could be interpreted as dimorphic, but identification of SSD and SBCD depended on which character was used as the reference (prosoma length, prosoma area, total length, principal component 1, or metasoma segment 1 width). Of these characters, discriminant function analysis suggested that metasoma segment 1 width was the most appropriate. The pattern of dimorphism in *H*. *arizonensis* mirrored that seen in other more obviously dimorphic scorpions, with static allometry trending towards isometry in most characters. Our findings are consistent with the conclusions of others that fecundity selection likely favors a larger prosoma in female scorpions, whereas sexual selection may favor other body parts being larger in males, especially the metasoma, pectines, and possibly the chela. For this scorpion and probably most other organisms, the choice of reference character profoundly affects interpretations of SSD, SBCD, and allometry. Thus, researchers need to broaden their consideration of an appropriate reference and exercise caution in interpreting findings. We highly recommend use of discriminant function analysis to identify the least-biased reference character.

## Introduction

The morphology of animals can be shaped by both natural selection and sexual selection [1,2]. Natural selection favors morphologies that enhance growth, reproduction, and survival, resulting in increased fitness for a given environment. Sexual selection favors morphologies that facilitate mating success via intrasexual competition intersexual mate choice, and post-copulatory success [3–5]. Sexual dimorphism—the different appearances of females and males of the same species—can arise from either of these adaptive processes, but it may also result from non-adaptive processes such as body-size scaling, genetic correlations between female and male body size, and phylogenetic constraints or inertia [6–9]. Sexual dimorphism can encompass an overall increase in size of one sex over the other (sexual size dimorphism, SSD), or it can be restricted to certain body parts, affecting their size, shape, or both (sexual body component dimorphism, SBCD). To distinguish between effects on overall size and effects on the size or shape of individual components (≈characters) that may or may not result in overall size differences, we introduce the latter term.

Dimorphism can also be considered from the perspective of allometry, as both often exist at the interface of natural and sexual selection. Allometry describes how body characters interact over the size range of an organism. Differential scaling of individual features results as selection favors either exaggerated (positive allometry) or reduced (negative allometry) size of some body components as body size increases, whereas others may remain proportional (isometry). Differences in scaling result from several interacting forces, including the physics of the structural shape in relation to the physical properties of the materials [10,11], and biological considerations of optimal use under natural selection with sometimes confounding effects of sexual selective pressures [12–14]. Whereas most characters follow negative allometry or isometry [15–17], characters shaped by sexual selection often exhibit strongly positive allometries [18–21]. However, the preponderance of sexual characters with positive allometries in the literature may be biased by extensive examination of exaggerated or extreme examples [16]. Indeed, a recent literature review demonstrated that many sexual signals, weapons, and other sexual traits exhibit isometry or even negative allometry. Thus, because positive allometry may actually occur in a minority of sexual traits, sexual selection alone may be insufficient to produce a positive allometric trend, and the presence of positive allometry may not be indicative of sexual selection [16,22–24].

A growing body of literature documents sexual differences in overall size and/or body component proportions of numerous animal species. This has certainly been the case for scorpions, although few authors have established a single measure or set of measures of overall body size, on average female scorpions show larger body sizes in terms of area or mass [25]; however, total length is often skewed toward males due to their often more elongate metasoma segments [26]. The exaggerated size of the pectines in males represents the most consistently dimorphic body component, resulting from an increase in both the number and size of the pectinal teeth [25]. Pectines comprise sensory organs that detect both physical [27] and chemical cues from the substrate [28–30]. The enhanced pectines of males are associated with mating, as they can follow the pheromonal trails laid down by females [31–33] and assess appropriate substrates for spermatophore deposition [34–38].

Several other body parts are frequently dimorphic in scorpions. The variably modified chelae structure of males [39–42] presumably aids in holding the female during the mating dance (*promenade aux deux*) [43,44]. The more elongate metasoma of males [45–48] potentially facilitates a sexual sting, fencing (*le arbre droit*), clubbing, and maybe even sexual identification while maintaining distance from a potentially aggressive female [25,49]. Sexual differences in prosoma and mesosoma size and shape may relate to the female’s role of producing and carrying offspring [50–54]. The functions of other occasionally dimorphic traits remain less clear, including differences in the telson and aculus shape [41,55], and in the presence of male accessory glands (e.g., subacular glands in several scorpion species [56,57] and the acular bulb in mature male *Anuroctonus* [58,59]).

Most studies that document scorpion dimorphism have reported differences in one or several body components, usually within the context of taxonomic descriptions. Often, the differences have been expressed by comparing the range of values for females and males, or the ratios for a single body part (e.g., length-to-width) to one or more other components (e.g., prosoma length, metasoma segment 5 length; [60]). Although these measures have their place in the literature, and greatly ease rapid identification of species or sex, they may lead to wrong inferences or spurious correlations [61], and cannot be used to discern which particular feature or body part might be under the influence of selection. When one sex is larger overall than the other, for example, differences in body components may simply reflect this SSD. And in the classic case for ratios, the conventional interpretation that sexual selection favors large male head size relative to overall length of lizards has been reinterpreted as fecundity selection favoring, instead, a larger trunk in females [62,63]. Thus, more refined approaches are required to understand the selective pressures that generate or maintain dimorphism, and even then differentiating the influences of natural and sexual selection on individual body components can be especially challenging [64,65].

Statistical methods such as analysis of covariance and regression are ideally suited for examining dimorphism and character scaling, as they can better normalize data, control for confounding variables, and are far more sensitive for evaluating subtle characters [66] that may still be under the control of natural or sexual selection. Potentially dimorphic characters or deviations from isometry are often identified by controlling for one body component, which acts as an overall indicator of general body size, followed by evaluation of how each body component of interest responds to changes in body size. The optimal scenario is to use a reference character that correlates with size, is independent of nutritional state[67], and is itself non-dimorphic[63]. However, the choice of an appropriate reference character can be fraught with difficulty [62,63,68–70], and may require the measurement of numerous body components. Choice of a reference character for body size can profoundly affect the assessment of dimorphism and its interpretation.

Here, we address the difficulties associated with measuring sexual dimorphism and character scaling through rigorous analyses of morphological variation in the desert hairy scorpion, *Hadrurus arizonensis*. Specifically, we used several alternative reference characters to evaluate SSD and SBCD for 16 morphological characters. We also assessed sexual differences in the static allometry of multiple body components to better understand their relationships to sexually dimorphic traits and the potential selective forces that shape them.

The desert hairy scorpion has long been viewed as non-dimorphic in characters other than the pectines [71,72]. Although Williams [56] mentioned that adult males have a longer metasoma than females, Stahnke [73] questioned the finding, and called for a more robust analysis beyond the raw data, including the use of ratios and statistical tests for comparison. Tallarovic [36] indicated there was no exaggerated dimorphism. While collecting specimens for other studies, one of us (GAF) became convinced that cryptic dimorphism existed in the species. The methodology presented here not only confirmed this suspicion, but should be useful for assessing sexual dimorphism and allometry in other scorpions. As our findings indicate for this scorpion, and probably for most other organisms, the choice of reference character can profoundly affect interpretations of SSD, SBCD, and the ways in which selection might act on these traits.

## Materials and Methods

### Ethics Statement

All methods in this study complied with the requirements of the Institutional Animal Care and Use Committee of Loma Linda University, which regulates animal research at this institution. At the time of the study, no protocol reviews or permits were required for any studies of invertebrates. However, the research met the ethical and academic integrity policies set forth by the Office of Research Affairs, and was reviewed and approved by the Faculty of Graduate Studies. This study also complied with federal and state laws, as *H*. *arizonensis* is not an endangered or protected species, and collections were made from public lands, where no permits or permissions were required for the activities performed.

### Scorpions

We collected adult specimens of *H*. *arizonensis* from the western Sonoran Desert between Cabazon and Whitewater, Riverside County, California, USA (33.898354, -116.682936: 33.910966, -116.651685). We captured them at night during the months of July to October using ultraviolet light sources [74]. We acquired a sample of 173 adult scorpions consisting of 82 males and 91 females (89.9–111.7 mm overall body length).

### Morphological measurements

**Fig 1:**
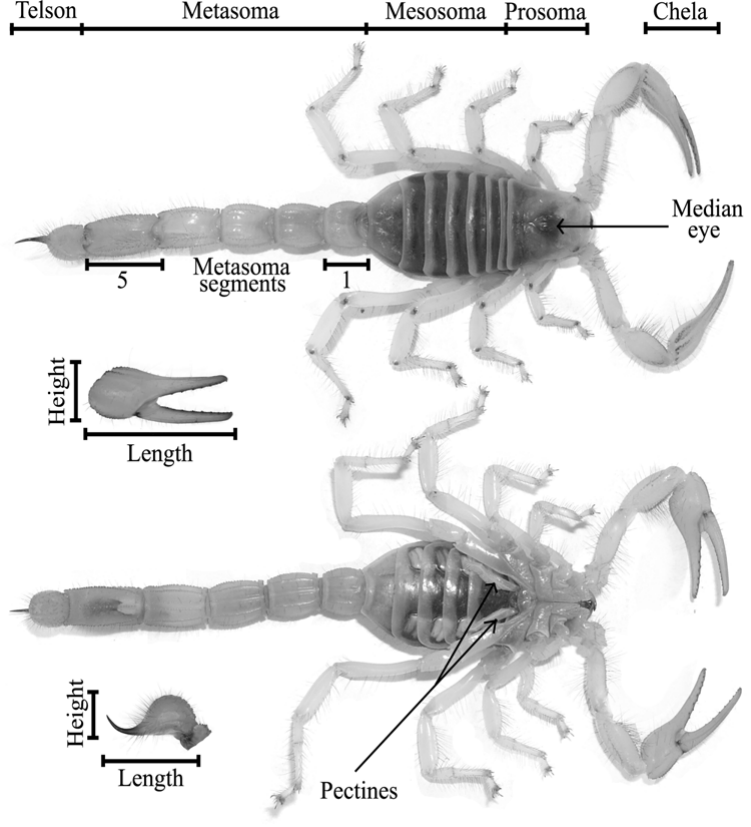
Morphology of representative Desert Hairy scorpion (*Hadrurus arizonensis*). Body components measured in this study are labeled.

Using electronic calipers, we measured to the nearest 0.1 mm the following characters (Fig. 1): total length (Tot L, edge of prosoma to end of metasoma); prosoma length (Pro L) and width (Pro W, at median eye); chela length (Chela L), width (Chela W), and height (Chela H); metasoma segments 1 and 5 length (Met 1 L, Met 5 L) and width (Met 1 W, Met 5 W); total metasoma length (Met L); length (Tel L), width (Tel W) and height (Tel H) of the telson; and pectine length (Pec L) [60]. We visually determined sex by relative length and arrangement of the pectines. We could have measured numerous additional characters reported in other studies (e.g., femur, patella, and other chela dimensions), but focused on what we believed were the most frequently reported dimorphic characters in scorpions. A secondary consideration was that the chosen measures could easily and reliably be done in the field for future comparisons. Although we measured mass and mesosoma size, we chose not to analyze these characters because both vary substantially with nutrition [67,75,76]. Taking measurements caused no apparent injury to the animals.

### Statistics

Prior to all statistical tests, we screened the data to verify compliance with parametric assumptions. We removed a small number of statistical outliers (studentized residuals >1.96) for specific body components while retaining other measurements of those individuals. Unless specified otherwise, statistical tests were conducted using SPSS 20.0 for Macintosh (Statistical Package for the Social Sciences, Inc., Chicago, 2011), with α = 0.05. Following Nakagawa [77], we chose not to adjust α for multiple tests. As an intuitive indicator for the magnitude of sex differences, we computed the percent difference for all characters analyzed (c.f. [78,79]) using the mean of each sex (i.e. [male—female] divided by 0.5 [male + female]).

We subjected the morphological measurements to five sets of analyses involving parametric tests [80,81]. Although pectine length and arrangement were used to determine sex, we elected to include Pec L in some analyses for comparative purposes, but omitted it from several analyses, as specified below.

First, we directly compared all body size components of females and males using independent-samples *t*-tests. We computed Cohen's *d* as a measure of effect size, with values of ~0.2, ~0.5, and ≥0.8 loosely corresponding to small, medium, and large effects, respectively [82]. Second, we employed discriminant function analysis (DFA) to determine which characters in multivariate space best discriminated between the sexes and those that were most neutral. We used an omnibus model including 14 variables (Pro L, Pro W, Chela L, Chela W, Chela H, Met 1 L, Met 1 W, Met 5 L, Met 5 W, Met L, Tel L, Tel W, Tel H, Tot L); the model excluded Pro A, a derived character which violated multicollinearity (tolerance = 0.00), and Pec L, which we used to determine sex. The DFA model was constructed with equal probability for group assignment and leave-one-out cross-validation. To determine the discriminating power of prosoma area, a second DFA was run which substituted Pro A for the components Pro L and Pro W. Following DFA, contrasts were conducted using ANCOVAs to determine which characters reliably separated the sexes after adjustment for the other characters or predictors [83]. In each ANCOVA, the variable of interest was declared the DV, sex was treated as a between-subjects factor, and the remaining characters were entered as covariates. Third, we conducted a principal component analysis (PCA) with Varimax rotation to evaluate covariance among the body size components and to create more general and uncorrelated measures of body size and shape. We excluded Pec L from the PCA model.

Fourth, we examined sexual dimorphism using five candidate reference characters via multiple analysis of covariance (MANCOVA) and analysis of covariance (ANCOVA) models. These models included sex as a between-subjects factor and one of the five covariates (reference characters) to control for overall body size. The covariates, tested in separate models, included Pro L, Pro A, and Tot L, as each has been used previously as an estimator of scorpion size and to evaluate sexual dimorphism [25,46,76,84]. We used principal component 1 (PC1) as the fourth covariate, which comprised a more general measure of body size based on multiple characters and has been recommended as a useful reference character for scaling [85,86]. Our fifth covariate, Met 1 W, was chosen because it contributed least to the discrimination between sexes in the DFA model. To our knowledge, no study has demonstrated dimorphism of this character in any scorpion. For MANCOVA and ANCOVA models, we always tested the assumption of homogeneity of regression slopes by including an interaction term, and then removed the term from the final model if the interaction was non-significant.

Finally, we used standard major axis (SMA) regression [87–89] to assess static allometry in females and males separately. Static allometry deals with comparisons among individuals in a population which are all at the same developmental stage, and can be distinguished from ontogenetic or developmental allometry, which makes comparisons across developmental stages either within the individual or at the population level [90]. We conducted bivariate analyses using the program SMATR [89], with α = 0.05, iterations (used for testing for common slope, Likelihood ratio test) = 10000, and H_0_ slope = 1 (*F*-test). We log_10_-transformed all variables including the square root of the prosoma area [46]. We compared the results from using four different reference characters to control for body size: Pro L, Pro A, Tot L, and Met 1 W. If male and female slopes were found to be the same, we conducted follow-up Wald tests to evaluate differences in elevation and shifts along the slope [87,89].

## Results

When morphological characters were considered individually via *t*-tests, adult female and male *H*. *arizonensis* exhibited sexual dimorphism in some but not all body components (Table 1). Females had significantly larger prosomas, averaging 2.06%, 1.52%, and 3.37% larger in length, width, and area, respectively. However, males had significantly larger Chela L (2.17%), Met 1 L (5.33%), Met 5 L (6.57%) and Met 5 W (1.74%), Met L (7.81%), Tot L (2.89%), and Pec L (17.09%). The remaining characters were not significantly different between the sexes (<1% difference).

**Table 1:** Comparison of morphological characters of adult female and male *Hadrurus arizonensis*.

Character	♀	♂	Mean		*t*—statistic	df	*P*-value	Mean Difference	Percent Difference (♂ to ♀)	Cohen's *d*
	(N)	(N)	♀	♂						
Pro L	90	83	13.1	12.9	-3.44	171	0.001	-0.3±0.1	-2.06	-0.52
Pro W	86	70	10.5	10.3	-2.07	154	0.04	-0.2±0.8	-1.52	-0.34
Pro A	86	70	137.5	132.9	-2.55	154	0.01	-4.6±1.8	-3.37	-0.41
Chela L	90	83	19.7	20.2	3.85	171	<0.001	0.4±0.1	2.17	0.59
Chela W	90	82	4.4	4.4	0.05	170	0.957	0.0±0.0	0.04	0.01
Chela H	86	70	6.9	6.8	-1.08	154	0.281	-0.1±0.1	-0.92	-0.18
Met 1 L	86	70	6.9	7.3	5.83	154	<0.001	0.4±0.1	5.33	0.94
Met 1 W	86	70	6.7	6.8	0.41	154	0.682	0.0±0.1	0.32	0.07
Met 5 L	86	70	12.8	13.7	8.66	154	<0.001	0.9±0.1	6.57	1.4
Met 5 W	85	69	5.8	5.9	2.48	152	0.014	0.1±0.0	1.74	0.41
Met L	87	77	47.7	51.6	10.75	162	<0.001	3.9±0.4	7.81	1.69
Tel L	89	83	12.8	12.8	0.06	170	0.952	0.0±0.1	0.04	0.01
Tel W	90	83	5.8	5.8	-0.49	171	0.625	0.0±0.0	-0.42	-0.07
Tel H	90	83	5.4	5.4	0.72	171	0.474	0.0±0.0	0.6	0.11
Tot L	89	79	100.6	103.5	4.12	166	<0.001	2.9±0.7	2.89	0.64
Pec L	85	68	10.1	12	18.8	151	<0.001	1.9±0.1	17.09	3.08

Mean (± 1 S.E.) difference between the sexes, relative to males (males larger if positive value; females larger if negative value)

The initial DFA model, which included 14 characters measured from 137 scorpions, confirmed that morphological differences between the sexes were highly significant (Wilks’ Λ = 0.12, χ^2^ = 266.87, df = 14, *P* < 0.001, canonical correlation = 0.936), with means for the discriminant function scores of-2.22 (range = -0.38 –-4.33) and 3.12 (range = 1.25–6.01) for females and males, respectively. Every scorpion (100%) was correctly assigned for both original and cross-validated classification. The three best discriminating characters were Met L, Met 5 L, and Pro L (standardized coefficients of 1.52, 1.10, and-1.03, respectively; all other characters 0.56; Table 2). Squared structure coefficients indicated that the function accounted for 12%, 7% and 1% of the variance in these characters, respectively. Signs for the function coefficients indicated that the difference between the sexes could largely be explained by the difference between metasoma length (represented by Met L and Met 5 L) and prosoma length, with males characterized by a longer metasoma relative to the prosoma. Contrasts using ANCOVA revealed that, after adjustment for all other predictors, only five characters provided significant discrimination between the sexes (listed in order of effect size): Met L (*P* < 0.001, partial η^2^ = 0.28; adjusted marginal means for females and males, 48.5 ± 0.2 and 51.0 ± 0.2 mm, respectively); Met 5 L (*P* < 0.001, partial η^2^ = 0.20; adjusted marginal means for females and males, 12.9 ± 0.1 and 13.6 ± 0.1 mm, respectively); Pro L (*P* < 0.001, partial η^2^ = 0.11; adjusted marginal means for females and males, 13.2 ± 0.04 and 12.8 ± 0.1 mm, respectively); Pro W (*P* = 0.005, partial η^2^ = 0.06; adjusted marginal means for females and males, 10.5 ± 0.05 and 10.2 ± 0.1 mm, respectively); and Tel L (*P* = 0.036, partial η^2^ = 0.035; adjusted marginal means for females and males, 12.7 ± 0.1 and 13.0 ± 0.1 mm, respectively).

**Table 2:** Standardized canonical coefficients of morphological characters of *Hadrurus arizonenesis* from two separate discriminant function analyses (DFAs).

Character	DF 1	DF 2
Met L	1.518	1.620
Pro A		-1.392
Met 5 L	1.099	1.091
Pro L	-1.025	
Pro W	-0.563	
Tot L	-0.354	-0.505
Tel L	-0.430	-0.399
Tel W	-0.323	-0.373
Met 5 W	0.308	0.369
Met 1 L	0.231	0.203
Chela W	-0.200	-0.219
Chela H	-0.196	-0.171
Chela L	0.147	0.078
Tel W	-0.125	-0.073
Met 1 W	0.020	-0.068

DF1: Discriminant function for DFA that excluded the character prosoma area due to multicolinearity

DF2: Discriminant function for DFA that excluded the characters prosoma length and width to test the influence of prosoma area

The second DFA model testing the influence of Pro A included 13 characters and was similarly significant (Wilks’ Λ = 0.13, χ^2^ = 265.75, df = 13, *P* < 0.001, canonical correlation = 0.935), with female and male discriminant function means of-2.20 (range = -4.36 –-0.15) and 3.09 (range = 1.09–5.92) respectively. Every scorpion (100%) was correctly assigned for both original and cross-validated classification. The three best discriminating characters were Met L, Pro A, and Met 5 L (standardized coefficients of 1.62, -1.39, and 1.09, respectively; all other characters ≤ |0.51| Table 2). Squared structure coefficients indicated that the function accounted for 13%, 1%, and 7% of the variance in these characters, respectively. As in the first model, signs on the discriminant function coefficients indicated that the difference between the sexes could largely be explained by the difference between metasoma length (represented by Met L and Met 5 L) and size of the prosoma (Pro A). Contrasts performed using ANCOVA revealed that, after adjustment for all other predictors, only Met L (*P* < 0.001, partial η^2^ = 0.33; adjusted marginal means for females and males, 48.3 ± 0.2 and 51.2 ± 0.2 mm, respectively), Pro A (*P* < 0.001, partial η^2^ = 0.24; adjusted marginal means for females and males, 140.5 ± 0.8 and 129.4 ± 1.1 mm^2^, respectively), Met 5 L (*P* < 0.001, partial η^2^ = 0.20; adjusted marginal means for females and males, 12.9 ± 0.1 and 13.6 ± 0.1 mm, respectively), and Tel L (*P* = 0.049, partial η^2^ = 0.031; adjusted marginal means for females and males, 12.7 ± 0.1 and 13.0 ± 0.1 mm, respectively) reliably separated the sexes.

In both DFA models, Met 1W was a poorly discriminating character (Table 2), and ANCOVA contrasts supported this conclusion (contrast following DFA model 1, *P* = 0.92, partial η^2^ = 0.001; contrast following DFA model 2, *P* = 0.74, partial η^2^ = 0.001). Thus, we considered Met 1 W to be the most suitable (i.e., most neutral) reference character, and added it to the remaining analyses.

The two principal components extracted from the PCA captured 77.4% of the variance (Table 3). The first (PC1), explaining 45.8% of the variance, was comprised largely of prosoma and telson size and shape, width of the two metasoma segments, and chela shape (width and height). The second (PC2), explaining 31.6% of the variance, included primarily overall metasoma length, length of the two metasoma segments, total length, and chela length. Females averaged significantly larger for PC1 (*t*_135_ = 5.36, *P* < 0.001, Cohen's *d* = 0.93), and significantly smaller for PC2 (*t*_135_ = 15.17, *P* < 0.001, Cohen's *d* = 2.65).

**Table 3:** Factor loadings for the two principal components (PC1, PC2) extracted from the principal component analysis of *Hadrurus arizonensis* morphological characters.

Character	Factor Loadings	
	PC1	PC2
Pro A	**0.916**	0.261
Pro W	**0.878**	0.245
Pro L	**0.874**	0.266
Tel W	**0.802**	0.294
Tel H	**0.774**	0.401
Met 1 W	**0.760**	0.407
Tel L	**0.753**	0.463
Chela H	**0.692**	0.351
Met 5 W	**0.643**	0.451
Chela W	**0.619**	0.445
Chela L	0.543	**0.725**
Tot L	0.543	**0.759**
Met 5 L	0.343	**0.880**
Met 1 L	0.287	**0.800**
Met L	0.216	**0.929**
Variance Explained (%)	45.8	31.6

**Fig 2:**
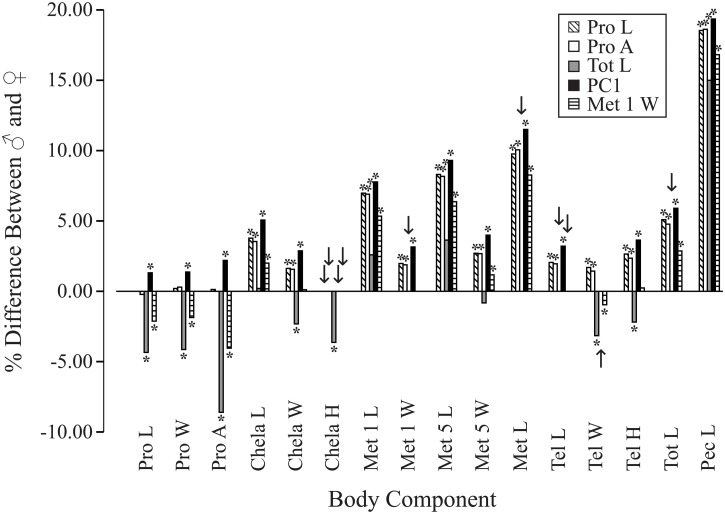
Sexual body component dimorphism (SBCD) in *Hadrurus arizonensis*, comparing the results of alternative reference characters. Analysis of covariance (ANCOVA) results are expressed as percent difference in marginal means between the sexes (y-axis) for each body component (x-axis groupings) when using different reference characters (covariates; indicated by bar pattern). Alternative reference characters included prosoma length (Pro L), prosoma area (Pro A), total length (Tot L), principal component 1 (PC1), and metasoma segment 1 width (Met 1 W). Percent difference was calculated as ((male marginal mean—female marginal mean)/((male marginal mean—female marginal mean)/2)) x 100. Thus, bars above zero indicate body components showing male-biased SBCD, and bars below zero indicate female-biased SBCD. Bars with an asterisk (*) indicate a significant difference between sexes. Missing bars (indicated by arrows) occur where a significant interaction between sex and the covariate (heterogeneous regression slopes) existed, precluding ANCOVA and obfuscating male-female differences. Note the incongruent interpretations of SBCD depending on which reference character is used in the ANCOVA. Additional details are provided in S1 Table.

The five characters selected for use as the reference or covariate for overall size in the MANCOVA and ANCOVA models (Pro L, Pro A, Tot L, PC1, and Met 1 W) provided incongruent results (Fig. 2, S1 Table). Use of Pro L, Pro A, and PC1 yielded largely identical interpretations (Pro L and Pro A both showing 12 of 15 characters dimorphic), with PC1 showing the greatest number of differences (14 of 16 characters dimorphic, and the other two characters displaying an interaction between sex and PC1). Most measures for the chela, metasoma, telson, pectine, and total length were substantially larger in males. Use of either Tot L (10 of 15 characters dimorphic) or Met 1 W (11 of 15 characters dimorphic) as the covariate indicated that females had significantly greater size for all prosoma measures. Remarkably, the dimorphism of some body components was reversed depending on which reference character was used. Prosoma characters were male-biased when PC1 was the reference and female-biased when Tot L and Met 1 W was the reference. Chela W was female-biased with Tot L as the reference, and male-biased with Pro L, Pro A, and PC1 as the reference. Telson W was female-biased with Tot L and Met 1 W as the covariate, and male-biased with Pro L and Pro A as the reference. Telson W was female-biased with Tot L as the reference, and male-biased with Pro L, Pro A, and PC1 as the reference. When multiple characters were combined in MANCOVA models, the results generally conformed with the ANCOVA models for individual characters.

A small number of interactions existed between sex and the covariate in the MANCOVA and ANCOVA models (14 of 99 models; 14.1%). In these models, the direction of sexual dimorphism could not be inferred because of a violation of the assumption of homogenous regression slopes. Detailed explanation of each interaction goes beyond our purposes.

**Fig 3:**
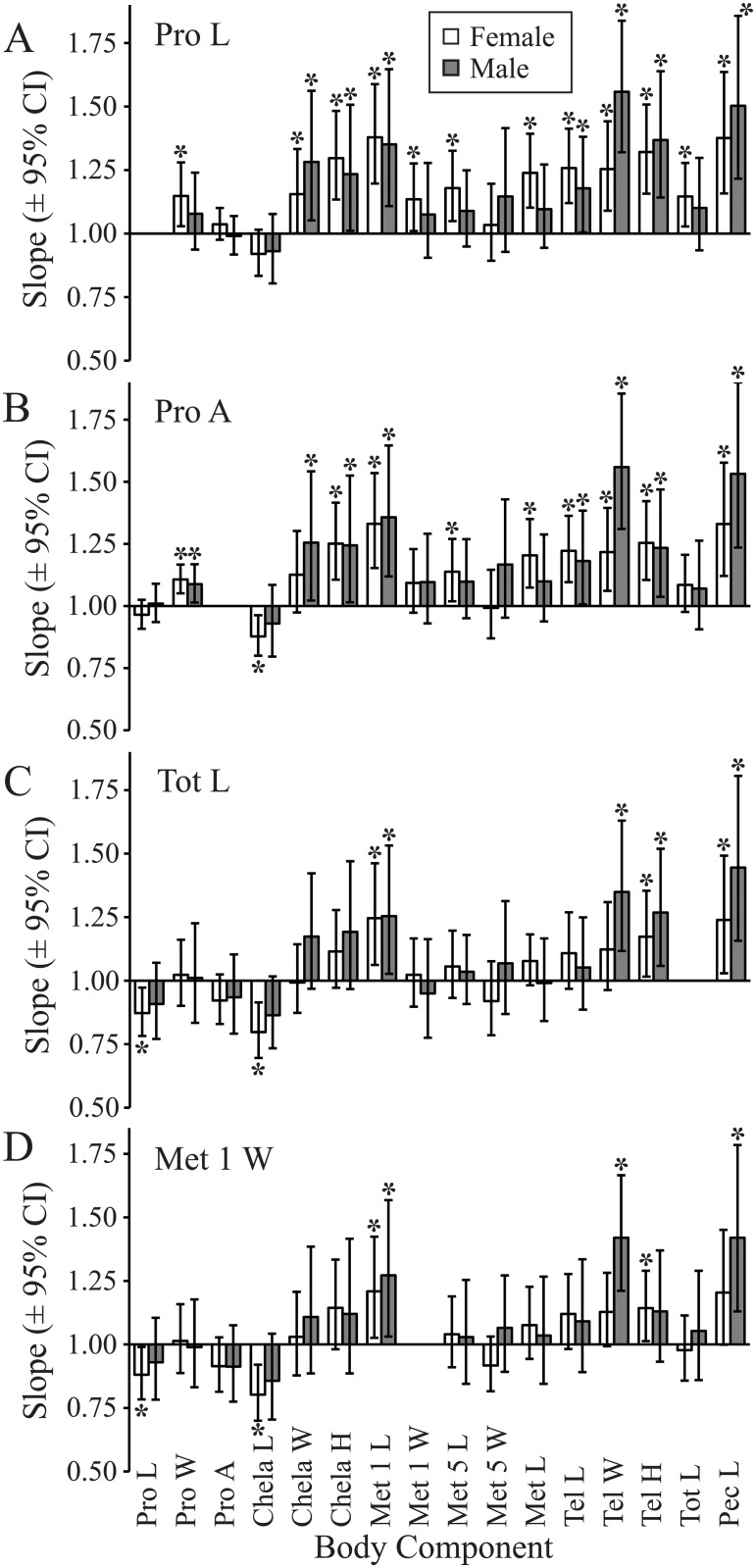
Effects of reference character on allometric trends of body components. Allometric slopes (± 95% CI) determined from four alternative reference characters are paired against each of 16 y-axis characters for females (N = 84–90) and males (N = 65–83). The reference characters included A: prosoma length (Pro L); B: prosoma area (Pro A); C: total length (Tot L); and D: metasoma segment 1 width (Met 1 W). Bars identified with an asterisk (*) indicate a significant difference between the slope and null hypothesis of 1.0 by *F*-test of standard major axis regression. Significant slopes above 1.0 indicate positive allometry; significant slopes below 1.0 indicate negative allometry; and non-significant slopes indicate isometry. Additional details are supplied in S2 Table.

**Fig 4:**
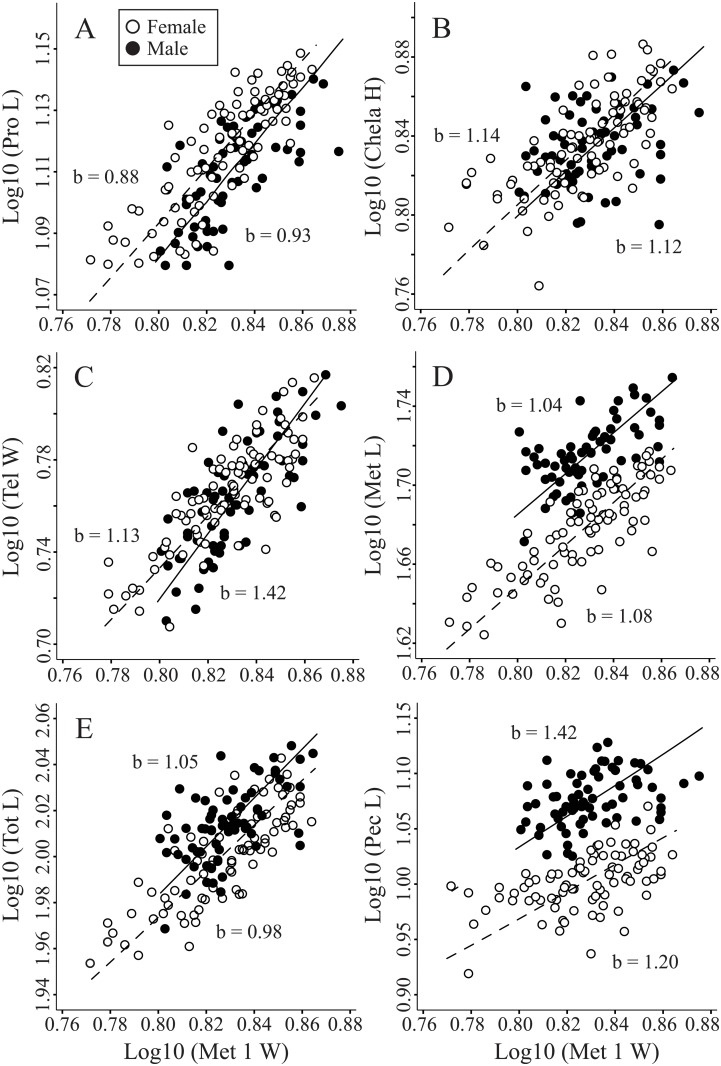
Select allometric relationships of female (open circles, dashed line) and male (closed circles, solid line) *Hadrurus arizonensis*. A–F depict static allometric scaling relationships of select body characters with metasoma segment 1 width (Met 1 W) as the reference character. A. Prosoma length (Pro L) plot illustrates a difference in y-intercept between the sexes. B. Chela height (Chela H) illustrates no difference between the sexes. C. Telson width (Tel W) illustrates a difference in slopes between the sexes. D–F Illustrate differences in both y-intercept and in shifts along the slope for metasoma length (Met L), total length (Tot L), and pectine length (Pec L). Scales are logarithmic. N = 84–90 females and 65–83 males. Additional details are supplied in S3 Table.

Based on SMA regression and SMATR output, we categorized allometric relationships (slope relative to 1.0) among the 16 body components and four reference characters as either positive, isometric, or negative. Allometric relationships were most often identical between the sexes, with only 28.3% of the models (17 out of 60) demonstrating a contrasting allometry (Fig. 3; S2 Table). Three body components (Pro A, Met 1 L, and Met 5 W) displayed the same allometry pattern across all four reference characters, whereas 13 body components showed contrasting allometries among the four reference characters. Prosoma L and Pro A as reference characters were similar to each other, showing congruent allometries for 9 of 14 body components. Total L and Met 1 W as reference characters were also similar to each other, yielding congruent allometries for 12 of 14 body components. However, allometric relationships derived from the two pairs of reference characters differed substantially from each other. Use of Pro L and Pro A as reference characters showed primarily positive allometry and isometry for both females (Pro L: 12 positive, 3 isometric; Pro A: 9 positive, 5 isometric, 1 negative) and males (Pro L: 7 positive, 8 isometric; Pro A: 8 positive, 7 isometric). In contrast, use of Tot L and Met 1 W as reference characters yielded comparatively more isometry and/or negative allometry for females (Tot L: 3 positive, 10 isometric, 2 negative; Met 1 W: 2 positive, 11 isometric, 2 negative) and males (Tot L: 4 positive, 11 isometric; Met 1 W: 3 positive, 12 isometric). Negative allometry was rare and only present for body components Pro L and Chela L in females. Although differences existed between sexes in designation of allometry as positive, isometric, or negative, only one body component differed statistically between the sexes in slope, and that was Tel W (Fig. 4D, S2 Table).

Representative comparisons in allometry between males and females for Met 1 W (the least biased) as the reference character are illustrated in Fig. 4 and summarized in S3 Table. Three body components (Pro L, Pro W and, Pro A) exhibited only a shift in elevation (y-intercept) between females and males. Seven body components (Chela L, Met 1 L, Met 5 L, Met 5 W, Met L, Tot L and, Pec L) showed a shift in both elevation and along a common slope. One body component (Tel W) showed a difference between slopes. Four body components were identical for the two sexes, showing no shifts in elevation or common slope (Chela W, Chela H, Tel L and, Tel H).

## Discussion

Although most scorpion species exhibit dimorphism in overall size (SSD) or individual body components (SBCD), the methods generally relied on to detect these (ranges in character measurements, ratios, and ANCOVA using a dimorphic reference character as a covariate) usually cannot identify which body parts are subject to selection. Here, we explored several candidate reference characters for overall body size to better understand sexual dimorphism and character scaling in a cryptically dimorphic taxon, *H*. *arizonensis*. We begin our discussion with general patterns of dimorphism, and then describe the dilemma of choosing an appropriate reference character for assessing dimorphism and allometry. We then consider sexual dimorphism and allometry of individual body components, and the selection forces that have potentially shaped them.

### General pattern of dimorphism

The most obvious conclusion from our analyses is that *H*. *arizonensis* could be interpreted as dimorphic in essentially every character. Simple *t*-tests demonstrated statistically significant dimorphism in multiple characters (10 of 16 measured; Table 1). Some characters had relatively small effect sizes (e.g., those of the telson), whereas others showed moderate (e.g., those of the Pro L and Chela L) or even large effect sizes (e.g., metasoma lengths and Pec L). However, univariate comparisons like these need to be viewed cautiously; if one sex is larger overall than the other sex, then a reference character for overall size needs to be controlled for. When controlling for overall size using ANCOVA, the identification of dimorphic body components varied depending on which character was used as the reference. With interactions included, 13 of 15 characters were dimorphic when each of Pro L, Pro A, Tot L, or Met 1 W was used as the covariate, and all 16 characters were dimorphic when employing PC1 as the covariate. Collectively, the ANCOVA models could suggest that every body component we measured is sexually dimorphic, even if most differences are quite small (<5%), i.e., cryptic. The fact that dimorphism exists at all in *H*. *arizonensis* has been largely overlooked by previous investigators [36,56,73].

### Choice of reference character and its implications

The choice of reference character or covariate for analysis of body component dimorphism varies widely among studies, and can substantially influence an assessment of dimorphism [69]. Most investigations rely on some measure of overall size as the covariate, or a proxy such as carapace width [91,92] or length [93], prothorax width [94,95], mass [96], total length [97,98], or snout-vent length [99,100], usually without offering justification. In each case, one can ask which is the target of selection: the reference character itself, the body component under consideration, or both? This problem was brought to the forefront recently by those studying lizards [62,63,68]. Previously, male-biased head size dimorphism was universally analyzed and interpreted using snout-vent length (SVL) as the reference character, and head size was considered the target of selection. Then the question arose as to whether selection was targeting the female's trunk (resulting in longer trunk via fecundity selection) or the male's head (resulting in larger size via sexual selection). As trunk length and head length are constituents of SVL, selection on either or both of these components could affect SVL, rendering SVL an inappropriate reference character. In spiders the common reference character is carapace width [92]. However, to study the comparative allometry of fang size in three spider species (*Scytodes thoracica*, *Loxosceles reclusa*, and, *Varacosa avara*), Suter and Stratton [70] opted to use sternum width as a proxy for size. The authors contended that use of carapace width was inappropriate, as it has been targeted by selection to a greater extent in *Scytodes* (indirectly due to venom gland hypertrophy [101]) than in other species. These examples illustrate the difficulties in choosing an appropriate reference character, the need to understand the organism of interest, and the potential for misinterpretation if these considerations are inadequately addressed.

Heretofore, scorpion sexual dimorphism and static allometry investigations have used several reference characters, including total length (*Bothriurus bonariensis*: [43]), prosoma + mesosoma (*Centruroides vittatus*: [49]), and prosoma area (*Centruroides margariatatus*: [46]). Studies of scorpion ontogenetic allometry and life history have utilized several prosoma measures [42,76,102,103]. We were initially interested in the use of a prosoma measure as a reference character in *H*. *arizonensis* due to precedent [92,104], its heavy loading on PC1 (c.f. [105]), and its avoidance of both the nutritional effects of the mesosoma and the frequent dimorphism present in the metasoma, each of which can influence total length. However, based on other scorpion species [41,45,106] and the findings of this study, the prosoma may itself be dimorphic, and therefore less than ideal [62,63]. Our DFA models support this conclusion, as prosoma variables had large unique contributions to each of the discriminant functions. We therefore considered body components that were poorly discriminating in the DFA models and demonstrated no dimorphism via *t*-test. Of the body components meeting these criteria (Chela W, Chela H, Met 1 W, Tel W, and Tel H), we propose Met 1 W as the best candidate reference character because it was the most neutral of all characters in the DFA models, and in contrast to other body components [39,41,59,107–109] has a high likelihood of neutrality in other scorpion taxa.

### Sexual size dimorphism (SSD)

The question of whether overall body size dimorphism exists in *H*. *arizonensis* remains unclear. Some body components were larger in females, and others were larger in males. When overall body size dimorphism was evaluated by examining whether the majority of individual body components showed female-larger dimorphism (negative percent difference, with most bars of a given color below zero line in Fig. 2) or male-larger dimorphism (positive percent difference), the direction of dimorphism shifted based on the reference character (covariate) employed. Using the prosoma (Pro L or Pro A) or PC1 as the reference character, the majority of body components averaged larger in males. However, Tot L as the reference character indicated the opposite situation, with most body components larger in females, excluding those commonly larger in male scorpions (Met 1 L, Met 5 L, Met L, and Pec L). Use of Met 1 W as the reference resulted in most characters averaging larger in males, and this would be our preferred interpretation, though it raises the question of which characters contribute most to overall size. Body mass would be an inappropriate measure of SSD because it is subject to nutritional and reproductive status.

Principal component 1 is a commonly used measure of body size in many taxa [85,86], and has been used as an indicator of overall size in scorpions [47]. In *H*. *arizonensis*, PC1 was positively and strongly associated with prosoma size, and averaged larger in females. However, interpretation of PC1 as a measure of overall size is complicated by the fact that it included characters representing both size (Pro L, Pro A, and Tel L) and, presumably, shape (e.g., Pro W, Tel W, Tel H, Met 1 W). Although PC1 is most commonly associated with size, it is not uncommon for both size and shape variables to load highly on a single component [86]. For example, variables representing both size and shape loaded highly on Graham et al.’s [47] PC1 used to differentiate scorpion species.

Considering the discordant measures of SSD, we cannot conclude which sex is larger overall. Nevertheless, we are confident that females have a larger prosoma and that males are longer overall (Tot L) due to their longer metasoma. These interpretations accord with the *t*-tests, ANCOVAs, and prior interpretations for scorpions in general [25,26].

#### Sexual body component dimorphism (SBCD), allometry, and potential selection

Our findings suggest that selection may act differently on the prosoma of *H*. *arizonensis* than on most of the body parts that extend from or beyond the prosoma, particularly the metasoma. The DFA models separated the sexes primarily on differences in prosoma variables (which loaded highly on PC1) and metasoma length variables (which loaded highly on PC2). In this section, we focus on inferences about individual body components. Although dimorphism (or the potential for dimorphism) is often noted in the scorpion literature (e.g., [25,41,46,55,110,111]), static allometry remains little studied in these taxa [43,46,49], and the use of different methods to analyze sexual dimorphism and allometry renders comparisons among studies problematic.

Female-biased dimorphism of the prosoma is consistent with the conclusion of others that fecundity selection has favored an increase in size of the prosoma of scorpion females compared to males that, along with the mesosoma, could support larger broods, larger offspring size, or both [50–54]. We therefore suggest that the prosoma should be avoided as a reference character for assessment of dimorphism and allometry in scorpions unless detailed analysis reveals it to be neutral for a given species. Isometry was the most common allometric trend for prosoma body measures across all reference characters in *H*. *arizonensis*, though some discordance existed. Using Met 1 W as the reference, the presence in females of negative allometry in Pro L and isometry in Pro W and Pro A suggests that the potential influence of fecundity selection may be constrained by other factors in this species.

Scorpions use their chela primarily to grasp items, particularly in predatory, defensive, and mating contexts, and therefore several interacting forces could influence selection on this body component. In *H*. *arizonensis*, choice of reference character confounded interpretation of SBCD for this particular body component, but male-biased chela length was apparent with Met 1 W as the reference character. Examples of both male-larger [111,112] and female-larger chela [41,113] can be found among scorpions, although evaluation of dimorphism using a neutral reference character could strengthen these interpretations. Chela are of utmost importance in prey capture and defense, to the point that envenomation is rarely or never used in adults of several scorpion species [114–117]. However, because diet and predators are presumably similar for the two sexes (to our knowledge these remain unstudied), we suggest that SBCD of this character in *H*. *arizonensis* may have arisen largely from either intrasexual or intersexual selection (c.f. [43]). Chela structure is important in mating behavior, and modifications of chela for this purpose have been suggested [39,41–44]. Female Chela L was the only character other than Pro L to display negative allometry (with Pro A, Tot L, or Met 1 W as reference), whereas Chela W and Chela H showed isometry with Met 1 W as the reference character (Fig. 3). Larger females may have disproportionately shorter chela in order to maintain the ability to interact efficiently with males during the *promenade aux duex* under a “one size fits all” model (sexual selection) [14,43]. As *H*. *arizonensis* relies largely, but not exclusively, on venom to obtain prey [118,119], natural selection may also act to increase relative Chela L in smaller females to enhance prey capture.

Although the metasoma is a prominent feature in scorpions, acting as the base and point of articulation for their venomous sting, the shape and structure of this tail can be variable both among species and between sexes [25]. Indeed, we found *H*. *arizonensis* males to possess a substantially longer metasoma (including segments 1 and 5) than females. Choice of reference character affected only degree of dimorphism for measures of metasoma length. Elaboration of the metasoma in the males of many species (e.g., [25,40,107,112,120] argues for a sexual role for this body segment, which could include combat with other males [49], clubbing or deflection of sting attempts by resistant females (e.g. [37,121]), and sexual stings toward females (e.g. [122,123]). A male-longer metasoma could alternatively be a by-product of different selection pressures on more sedentary females compared to more vagile males [121,124,125], resulting in different foraging [126] or defense/escape [49] tactics. We suspect that sexual selection has shaped the dimorphism of this body component in *H*. *arizonensis*, but more study is needed. The direction of allometry in metasoma body components was largely similar across all references and isometry was the dominant trend. Positive allometry was present for Met 1 L for both sexes across all references. As the first metasoma segment is the connection between the scorpion body and tail, a disproportional increase in the size of this segment in larger individuals may be related to mechanical constraints.

Variation in telson morphology by species and sex has been described [25,55,59,127], but explanations invoking functional relationships seem to be absent. Interpretations of dimorphism differed depending on reference character, but subtle female-biased dimorphism (Tel W) existed with Met 1 W as the reference character. As the telson harbors the venom glands and the musculature that controls venom expulsion, SBCD in this structure could have important implications for possible sexual differences in venom availability and use. Scorpions (with a few exceptions) rely on their venom not only for predation and defense [119,128,129], but males may also use their venom in a sexual sting, which has been described in *H*. *arizonensis* [122]. Stabilizing selection nevertheless may be acting on the telson to optimize venom supply for both sexes. Telson characters were generally isometric or positively allometric (Tel H for females, Tel W for males) with Met 1 W as the reference. Larger scorpions tend to possess disproportionately larger telsons, but predominantly isometrically-scaled chela suggests a consistent reliance of *H*. *arizonensis* on venom rather than chela for subjugation of prey. It would be interesting to compare allometry of the telson and chelae in *Pandinus imperator*, which uses venom to subdue prey when young, but relies primarily on the chelae as adults [114].

Variation in pectine size and structure may be the best characterized SBCD, as it is unique to scorpions and often relied upon by investigators to determine sex. Our pectine-related results align well with findings from other species: females had smaller pectines than males, and the pectines were the most dimorphic character by *t*-test and in all ANCOVA models. Pectines function to identify physical [27] and chemical cues [28] on the substrate, which enable pheromonal sex discrimination [32], mate trailing [29,32], and spermatophore deposition [34,35,121], suggesting a strong influence from sexual selection. Intersexual and interspecific differences in pectine structure may reflect, for example, differing degrees of vagility in scorpions. Males typically travel more and occupy larger home ranges than females, particularly during the breeding season when males are searching for mates, and given the sensory importance of the pectines, exaggeration of this body component in males is reasonable [58,124,130–132]. However, the pectines may also function in prey detection [133,134], and therefore could be under the influence of natural selection. Positive allometry with Met 1 W as the reference character (significant for males and approaching significance for females) similarly suggests selection arising from the functional roles of pectines in adults.

## Conclusions

In our attempt to statistically characterize cryptic sexual dimorphism and character scaling in *H*. *arizonensis*, we encountered serious difficulties in finding a suitable reference character for overall body size. Of the reference characters we examined (Pro L, Pro A, Tot L, PC1, and Met 1 W), the prosoma-based characters and PC1 are likely poor choices in this species, as they are all dimorphic measures, and the prosoma characters contributed unique variance within DFA models sufficient to differentiate the sexes. Although Tot L was also dimorphic, it was a poorly discriminating character in DFA models, and therefore potentially a better choice of reference for *H*. *arizonensis*. We selected Met 1 W as the best reference character however, as it was the most neutral of all characters examined. We suspect that Met 1 W as a reference has the greatest likelihood of utility in other scorpion taxa, as Tot L and the other body components evaluated often demonstrate greater dimorphism in other taxa than in *H*. *arizonensis*.

The direction of dimorphism in *H*. *arizonensis* for most characters mirrored that seen in other more obviously dimorphic scorpions. Our findings are consistent with the conclusions of others that fecundity selection likely favors a larger prosoma in female scorpions, whereas sexual selection may favor other body parts being larger in males, especially length measures of the metasoma, pectines, and possibly the chela. While we expected most characters to be isometric in *H*. *arizonensis*, we were surprised by both the negative allometry of Pro L (female) and positive allometry of Met 1 L (both sexes). As methodology for evaluating static allometry is still being established for scorpions, interspecific comparisons await future study.

For *H*. *arizonensis*, and probably for most other organisms, the choice of reference character can profoundly affect interpretations of SSD, SBCD, and allometry. Thus, researchers need to broaden their consideration of an appropriate reference, and exercise more caution in interpreting their findings, especially as they relate to selection. We highly recommend use of discriminant function analysis as a useful means for identifying the most appropriate (unbiased) reference character. Further studies including more species and a wider range of morphological characters will shed further light on our understanding of sexual dimorphism and character scaling in scorpions.

## Supporting Information

S1 Dataset Raw data measurements of body components in *Hadrurus arizonensis*. A small number of statistical outliers (studentized residuals >1.96) were removed for specific body components while retaining other measurements of those individuals.
(XLSX)

S1 Table Comparison of marginal means (± 1 S.E.) for morphological characters (dependent variables, DVs) of adult female (N = 84–90 for each character) and male (N = 65–83 for each character) *Hadrurus arizonensis* from MANCOVA (grey shading) and ANCOVA models using four alternative reference characters (covariates) to assess dimorphism. Analyses conducted using untransformed data.
(XLSX)

S2 Table Comparisons of static allometry for morphological characters of adult female (N = 83–91) and male (N = 64–81) *Hadrurus arizonensis* from standard major axis regression models using four alternative reference characters. Slope values of each sex were compared to the theoretical isometric value of 1.0. The male and female slopes were then compared to each other for each morphological character, testing for commonality of slopes and elevations (y-intercept) between the sexes.
(XLSX)

S3 Table Comparisons of static allometry for morphological characters of adult female (N = 83–91) and male (N = 64–81) *Hadrurus arizonenesis* from reduced major axis regression models using 4 alternative reference characters. Slope values of each sex were compared to the theoretical isometric value of 1. The male and female slopes were then compared to each other for each morphological character, testing for commonality of slopes, and elevations between the sexes.
(XLSX)
